# A physiologically-based pharmacokinetic model of oseltamivir phosphate and its carboxylate metabolite for rats and humans

**DOI:** 10.5599/admet.628

**Published:** 2019-02-23

**Authors:** Guanghua Gao, Francis Law, Ricky Ngok Shun Wong, Nai Ki Mak, Mildred Sze Ming Yang

**Affiliations:** 1 Center for Drug Evaluation, China Food and Drug Administration, Beijing 100022, China; 2 Department of Biological Sciences, Simon Fraser University, Burnaby, B.C. Canada V3N 4W8; 3 Department of Biology, Hong Kong Baptist University, Kowloon, Hong Kong. SAZ. China

**Keywords:** Oseltamivir, physiologically-based pharmacokinetic modelling, tissue concentration, dose metrics

## Abstract

Oseltamivir phosphate (OP, Tamiflu®) is a widely used prodrug for the treatment of influenza viral infections. Orally administered OP is rapidly hydrolyzed by the carboxylesterases in animals to oseltamivir carboxylate (OC), a potent influenza virus neuraminidase inhibitor. The goals of this study were to develop and validate a physiologically-based pharmacokinetic (PBPK) model of OP/OC in rats and humans, and to predict the internal tissue doses for OP and OC in humans after receiving OP orally. To this end, a PBPK model of OP/OC was first developed in the rat, which was then scaled up to humans by replacing the physiological and biochemical parameters with human-specific values. The proposed PBPK model consisted of an OP and an OC sub-models each containing nine first-order, flow-limited tissue/organ compartments. OP metabolism to OC was assumed to carry out mainly by hepatic carboxylesterases although extra-hepatic metabolism also occurred especially in the plasma. The PBPK model was developed and validated by experimental data from our laboratories and from the literature. The proposed PBPK model accurately predicted the pharmacokinetic behavior of OP and OC in humans and rats after receiving a single or multiple doses of OP orally or an OC dose i.v. The PBPK model was used to predict the internal tissue doses of OP and OC in a hypothetical human after receiving the recommended dose of 75 mg/kg OP b.i.d. for 6 days. Steady-state OC concentrations in the plasma and major organs such as the lung and the brain were higher than the minimum in vitro IC50 reported for H1N1 influenza virus neuraminidase, confirming OP is an effective, anti-viral agent. OP side-effects in the gastrointestinal tract and brain of humans were explainable by the tissue doses found in these organs. The PBPK model provides a quantitative tool to evaluate the relationship between an externally applied dose of OP and the internal tissue doses in humans. As such the model can be used to adjust the dose regimens for adult patients in disease states e.g., renal failure and liver damage.

## Introduction

**Figure 1.**
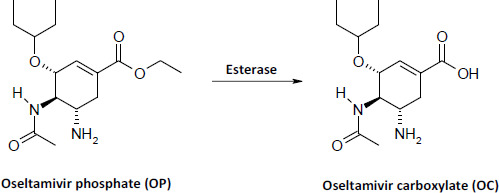
Hydrolysis of oseltamivir phosphate to oseltamivir carboxylate

Oseltamivir phosphate (OP, Tamiflu®) is an effective prodrug for the prevention and treatment of influenza viral infection [1]. After oral dosing, OP is rapidly absorbed into the systemic circulation of humans and hydrolyzed by the carboxylesterase enzymes in different tissues and organs to oseltamivir carboxylate (OC), a potent inhibitor of the neuraminidase enzyme [2] (Fig. 1). Because influenza virus needs neuraminidase to grow and duplicate, OC is able to reduce the duration and severity of influenza viral infection in humans [3].

OP is a drug with a wide margin of safety. However, adverse events such as abdominal pain, diarrhea, nausea, and skin reaction have been reported during early medication periods especially if the oral dose is >200 mg OP *b.i.d*. [3,4]. Thus neurological symptoms such as headache, vertigo, somnolence, insomnia, numbness, hyper-excitation and an increase in suicidal tendency also have been reported in Japanese children and young adolescents [5,6]. The mechanism(s) by which OP causes neuropsychiatric effects remains unclear: Toovey et al. [7] have suggested that since OP and OC are P-glycoprotein substrates [8], little or no OP/OC is able to penetrate the blood-brain barrier of humans and cause neuropsychiatric effects [9]. Conversely, OP has been shown to block ganglioside-regulated excitatory opioid receptor-mediated hyperalgesia and increase opioid analgesia in mice [10,11]. Thus, OP or OC may alter the neuronal excitability of the brain by increasing the synchronization of hippocampus network [12]. It is also possible that OP/OC change human behaviors if the blood-brain barrier is immature or damaged as in the case of young children.

OP metabolic disposition has been studied in rats [13], ferrets [14] and mice [15]. The oral bioavailability of OP in these animals ranges from 30-73 % [16] but in humans, it may reach 80 %. Therapeutic levels of OC have been found in the lungs, trachea, nasal mucosa of ferrets [14], and the middle ear and sinuses of humans [17]. However, little or no information is available on the dose metrics of OP and OC in the internal tissues/organs of humans. Andersen [18] has suggested using the physiologically based pharmacokinetic (PBPK) model to predict the pharmacokinetics and tissue doses of environmental chemicals in humans. Moreover, by determining the relationship between the internal tissue dose (instead of the external dose) and the pharmacological or toxicological effects of xenobiotics, we can greatly improve the accuracy of the dose-response relationship of prescription drugs and chemicals [19].

Hitherto, at least two different OP PBPK models have been reported for neonates, infants and adult humans [20,21]. None of these models have been used to predict an internal tissue dose of OP/OC in humans. Parrott et al. [20] first develop an OC PBPK model in marmoset monkeys and then extrapolate the model to young children. The PBPK model was developed using a multi-compartment permeability-limited liver. Due to a lack of tissue distribution data in monkeys and humans, the PBPK model needs refinement and improvement.

The aims of this study were to develop and validate a PBPK model for OP/OC in rats, to scale up the rat model to humans, and to predict the internal tissue doses of OP/OC in rats and humans using peak concentration (*C*_max_) and/or area under the curve (AUC_plasma_) of concentration-time curve as the dose surrogates. Attempts were made to explain the therapeutic and/or side effects of OP in humans using the model-predicted dose metrics. Since our OP/OC PBPK model is based on the actual anatomy and physiology of rats (or humans) and the physicochemical properties of OP/OC, it should be useful in predicting the internal tissue doses of OP/OC across the external doses, administration routes, and animal species [22].

## Materials and methods

### Metabolic disposition of OP in the rat

#### Chemicals

OP/OC standards were kindly donated by Hoffmann-La Roche Ltd., Switzerland. Chemical purity of both chemicals exceeded 99 %. Dichlorvos was purchased from Supelco (Bellfonte, PA, US). Analytical grade diethyl ether and HPLC grade acetonitrile were purchased from Labscan Asia Co. Ltd (Bangkok, Thailand).

#### Animals

Male Sprague-Dawley rats weighing about 250 g were purchased from the Chinese University of Hong Kong, Hong Kong. The animals were maintained on a constant light-dark cycle with light from 07:00 to 19:00 h and darkness from 19:00 to 07:00 h. Tap water and food were provided ad libitum. The rats were used after a 7-day acclimation period. The procedure associated with animal care and experimentation was carried out according to the Hong Kong Council on Animal Care guidelines and with formal approval of the Animal Care Committee of the Hong Kong Baptist University.

#### Tissue distribution studies

Thirty rats were used in the tissue distribution studies. The rats were assigned randomly into two groups of 15 rats each. After fasting overnight, one group of rats were given a single dose of 10 mg/kg OP by gavage. The other group of rats received 50 mg/kg OP. Three rats from each group were selected randomly and euthanized by diethyl ether at the following time points: 0.5, 2, 3, 6, and 12 h post-dosing for the 10 mg/kg dose group; 0.5, 2, 3, 6 and 10 h post-dosing for the 50 mg/kg dose group. The abdominal wall was opened and a blood sample was withdrawn by cardiac puncture. The blood samples were centrifuged immediately at 3000 rpm for 10 min to collect the plasma. The plasma was put in a centrifuge tube containing a mixture of sodium heparin and dichlorvos which was used to inhibit OP hydrolysis in the sample [23]. The liver, spleen, lung, and kidney were also removed from the rats. The tissue samples were rinsed briefly with distilled water, wiped dry with Kimwipes, and stored at -80 °C until analysis.

#### Extraction of OP and OC from plasma and tissue samples

One gram of tissue/organ (0.5 g for spleen) was weighed accurately in a test tube. After adding distilled water (3 ml) and dichlorvos (600 μg), the tissue/organ was homogenized in a Kinemetica GmbH homogenizer (PCU-2-110, Switzerland) for about 2 min. A 0.2 ml aliquot of the tissue homogenate (or plasma) was added to 0.8 ml of acetonitrile/water (3:1, v/v) in an Eppendorf tube. The content in the Eppendorf tube was mixed by vortex for 1 min and then centrifuged at 13000 rpm for 10 min. The supernatant was removed and stored at -80 °C until analysis.

#### LC/MS/MS analysis of OP and OC

Frozen tissue extracts were thawed, diluted with the mobile phase, and injected 10.0 μl of the final solution into the HPLC. OP/OC in the extracts was quantified using a modified LC/MS/MS procedure reported by Lindegardh et al. [24]. Briefly, OP/OC separation was performed on a C18 analytical column (ODS-2, 150mm, 5u, Waters Corp. Milford, MA) in a HP 1100 system (Hewlett Packard, Santa Clara, California) preceded by a guard column (2.1 by 5 mm; particle size, 1.7 μm) (Waters Corp. Milford, MA). The column was maintained at 26 °C; it was eluted isocratically at a flow rate of 0.200 ml/min. The HPLC mobile phase consisted of a solution with an equal volume of ammonium formate (8.0 mM) and methanol. The solution was adjusted to pH 3.50 with formic acid. The analysis employed an API 3200TM triple quadrupole mass spectrometer (AP Scienx) equipped with a Turbo VTM ion source controlled by the LINAC® collision cell technology for output scanning. The optimal transitions were 313.14 m/z to 224.92 m/z for OP, 285.2 m/z to 197.0 m/z for OC. The limits of detection for OP and OC were 20 and 30 pg/ml, respectively. The limits of quantification ranged from 0.4-0.6 ng/ml for plasma and 1.6-2.4 ng/g for tissues. Recoveries were achieved by comparing OP/OC concentration in the extract to the theoretical concentration; they ranged from 96 % to 101 % in the plasma/tissue samples. No obvious matrix effects were detected in the tissues within the OP/OC concentration ranges used in this study.

#### Blood to plasma ratio

About 2 ml of freshly drawn rat blood was mixed with a solution containing of 100 μg/ml OP (or OC) standard and 200 μg/ml of dichlorvos. Preliminary studies indicated that equilibrium between the plasma and blood for OP/OC was established within 20 min. After incubating the whole blood at 37 °C for 30 min, an aliquot was removed and centrifuged at 3000 rpm for 10 min to obtain the plasma. OP and OC concentrations in the plasma were measured by LC/MS/MS as described above. OP and OC concentrations in the blood were assumed to be the theoretical concentrations. Blood/plasma concentration ratio (BLPLR) was calculated by dividing the concentration of OP (or OC) in the whole blood with that in the plasma. The BLPLR of OP and OC was about 1.0 which indicated OP and OC had similar protein binding capacities to blood and plasma.

#### OP metabolism by rat plasma *in vitro*

In vitro OP metabolism was studied by incubating different concentrations of OP solution (0.1-0.5 mg/ml) with 30 μl rat plasma and Tris-HCl buffer (pH 7.4) in a shaking water bath maintained at 37 °C. The final incubation volume was 200 μl. Control incubation was carried out in a similar manner but without rat plasma. At 1 h after incubation, ice-cold acetonitrile (400 μl) was used to terminate the hydrolysis reaction. The mixture was centrifuged at 13200 rpm in a 4 °C centrifuge (Model 5415R, Eppendorf AG, Barkhausenweg 122339 Hamburg, Germany) for 10 min to separate into layers. The supernatant was transferred to an HPLC vial and analyzed by a Hewlett Packard 1100 HPLC equipped with a UV detector. OP and OC were separated on an HPLC column (ODS-2, 150 mm, 5 μm) using gradient elution. The mobile phase consisted of a solution of 0.4 % phosphoric acid (pH 3.0) and acetonitrile (80:20, v/v). Acetonitrile in the mobile phase increased linearly from 20 % to 40 % in 10 min. The flow rate of the mobile phase was 1 ml/min. The detection wavelength was set at 215 nm. OP metabolism rate was expressed as μg OC formed/ml plasma/h. The *V*_max_ and *K*_m_ of OP metabolism were determined using the Lineweaver-Burke plot and were found to be 61.2 mg/h and 300 mg/L, respectively.

### PBPK modeling of OP/OC in rats and humans

#### Model structure

**Figure 2.**
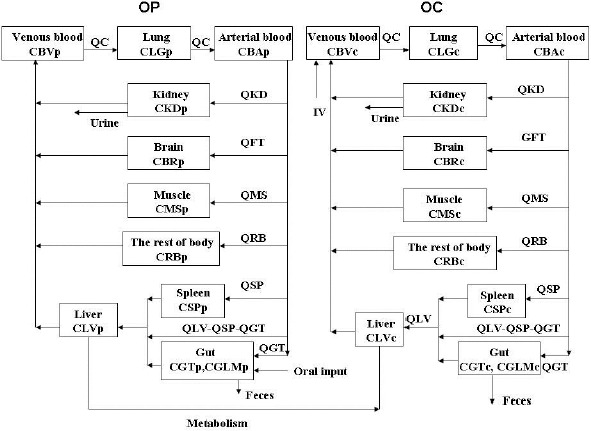
Schematic diagram of the physiologically-based pharmacokinetic model for rats or humans after OP administration. C represents OP or OC concentrations (ng/g or ng/ml); Q represents plasma flow rates (l/h). The subscripts (p or c) under specific tissue concentration C in the sub-models refer to OP or OC, respectively

Fig. 2 shows a schematic diagram of the OP/OC PBPK model in rats after oral administration. The PBPK model actually consisted of an OP sub-model and an OC sub-model. Each sub-model was composed of 9 first-order flow-limited tissue compartments: the lung, kidney, muscle, brain, liver, spleen, gut, blood and rest of body. The brain was lumped with the fat and the lung was lumped with the heart because of possible similar tissue/plasma partition coefficients. The rest of body compartment included all other tissues which had not been identified in the model *i.e.*, the skin, bone, eye, prostate gland, *etc*. The lung and the brain are modeled as separate compartments because H5N1 influenza virus mainly affected the respiratory tract and the central nervous system of Japanese children after oral administration of OP [5,6]. Absorption of OP was assumed to be a linear, first-order input process. After absorption, OP was hydrolyzed to OC mainly by the carboxylesterases in the plasma and the liver. For the purpose of model development, OP metabolism was assumed to occur only in the liver which linked the OP and OC sub-models together (Fig. 2). Also, one mole of OP was converted by the carboxylesterase enzymes to one mole of OC. Both OP and OC were excreted into the urine by the kidney.

#### Physiological parameters

The physiological parameters of the rat PBPK model were parameterized *a priori* (Table 1) as follows: tissue volume and blood flow were taken from Luttringer et al. [25] and from Davies and Morris [26]; these parameter values were expressed as the percentage of total body volume or cardiac output (CO). Total blood volume was divided into a two-thirds venous pool and one-third arterial pool. Gut content volumes was assumed to be 0.014 L for a 0.25 kg rat [27].

#### Pharmacokinetic parameters

Absorption rate constant (*k*_a_) of OP was determined to be 0.76 h^-1^ which was obtained by fitting the OC concentration-time curve in plasma following oral administration of 10 mg/kg of OP to a classical, one-compartment pharmacokinetic model. Considering the uncertainty of the absorption related parameters, *F* and *K*_a_ were allowed to be optimized to a reasonable extent during the modelling process based on the individual experimental datasets (Table 2). The oral bioavailability of OP was reported to be 0.35 calculated by AUC_p.o._ (OP)/AUC_i.v._(OC) ratio, it should be noticed that in the PBPK model *F* represents the oral bioavailability factor which was derived from the integrative modeling process of the PBPK model for all organs [28] and might be different from the experimental absolute bioavailability (AUC_p.o._/AUC_i.v._). The clearance (CLc) of OC from rats was reported to be 0.38 L/h after injecting a single dose of 10 mg/kg intravenously [15,16]. *In vitro K*_m_ (300 mg/L) and *V*_max_ (61.2 mg/h) values were obtained by incubating OP with rat plasma. Fecal excretion rate constant (*k*_f_) was assumed to be 1/transit time of the small intestine; the transit time of the small intestine was taken from Davies and Morris [26].

#### Tissue/plasma partition coefficients

These were estimated by applying the area method of Gallo et al. [29] to the experimental data of rats after receiving a single oral dose of 10 mg/kg OP (Table 1). Briefly, the experimental OP/OC concentration-time curves in the plasma and organ/tissue were analyzed separately with the non-compartmental approach (WinNonlin®, Scientific Consulting, Inc. Version 1.5) to obtain the area under the concentration-time curve (AUC). The *in vivo* tissue/plasma partition coefficient for a specific organ was estimated from the AUC_tissue_/AUC_plasma_ ratio, Table 1, lists the final tissue/plasma partitioning coefficients which had been adjusted to available experimental data. In rat, plasma protein binding for OP was reported to be 53.7 % [30], similar binding capacity in blood as in plasma was found in our study for both OP and OC. The tissue/plasma partition coefficients basically agreed with the lipid contents of the tissues and the lipid solubility of OP or OC.

#### Model simulation

The differential and algebraic equations describing the movement of OP and OC through the rat or human were formulated as a computer program. Mass balance differential equations for the model and the definition of the algebraic terms are given as parts of the model structure (Fig. 1 and Appendix). After incorporating the parameter values (Tables 1 and 2) into the model (Fig. 1), the differential and algebraic equations were solved numerically with the aid of AcslXtreme®2.4.2.1 (Aegis Technologies Group, Inc., Huntsville, AL). An *i.v.* injection function instead of oral absorption was used to drive the model when OP/OC was administered intravenously [31].

#### Scaling up of rat PBPK models to humans

The rat model (Fig. 2) was scaled up to humans by replacing the physiological parameters and pharmacokinetic parameters in the rat model with human specific values (Table 1). The human PBPK model was parameterized as follows: (a) tissue volumes and blood flows to tissues were taken from the literature [25,26], (b) tissue/plasma partitioning coefficients were assumed to be the same as those of rats, (c) *F* was set to 75 % based on the absolute bioavailability reported in humans [14,32], (d) plasma protein binding of OC and OP in humans was 3 % and 43 %, respectively [4], (d) gut content volumes was scaled based on the body weights of rats and humans; the gut content of a 70 kg human was determined to be 0.16 L [26], (e) clearance of OC (Cl_c_) in urine was reported to be 18 L/h [14,33]. Clearance value for OP (Cl_p_) was scaled from the clearance of rats using the allometric equation, CL_bc_ = CL_b_/(BW)^0.66^ [34] initially, it was reported to be 20 L/h while CL/*F* was 0.438 L/min [14], considering the uncertainty of absorption parameters and the difference between classical model and PBPK model, Cl_p_ was allowed to be optimized using the plasma concentration-time curve reported by He et al. [14], (f) *in vitro K*_m_ and *V*_max_ of OP metabolism in human liver microsomal incubates were reported to be 187 μM and 114 nmol/mg/min, respectively [2]; these translated into in vivo *K*_m_ and *V*_max_ values of 76 mg/L and about 2.6x10^5^ mg/h, respectively for humans based on an average microsomal yield of 52.5 mg microsomal proteins per g liver [14,31,35], and (g) *k*_a_ and *k*_f_ rate constants (Table 2) were scaled up from rats using the equation, *k*_c_=*k*_a_/(BW)^-0.3^, where *k*_a_ represented the rate constant, *k*_c_ represented the scaling coefficient and BW was the average body weight of human volunteers [36]. Model parameters that could not be parameterized *a priori* were optimized by fitting the PBPK model to available experimental data (see Data fitting below). Final parameter values used to implement the human PBPK model are listed in Table 3.

#### Data fitting and parameter optimization

The parameters that could not be obtained *a priori* or determined accurately were optimized by fitting the PBPK model to available data. Optimization was accomplished by minimizing the squared differences between model prediction and the experimental data using the maximized log likelihood function of the AcslXtreme® OptStat program (Aegis Technologies Group, Inc., Huntsville, AL). Only the parameters that needed to be parameterized were varied, the values of other parameters in Tables 1 and 2 were kept constant.

#### Validation and calibration of PBPK model

The PBPK models of rats and humans were validated or calibrated by comparing model-simulated results with experimental data not used in model development. The empirical data were obtained either from our own laboratories or from the literature. Because we were unable to obtain the original concentration-time data from the literature, they were read digitally from the publications using DigiMatic® (Windows version 2.2c, FEB Software, Chesterfield, Virginia).

The PBPK model was assumed to be validated if model simulation described closely the experimental data. Thus, the rat PBPK model was validated using the experimental data from rats dosed with a single oral dose of 50 mg/kg OP. The rat model was further validated using the plasma concentration-time data from rats after receiving a single oral dose of 30 mg/kg OP or an *i.v.* infusion of 30 mg/kg OC [37]. Similarly, the human PBPK model was calibrated using the datasets reported by He et al. [14] for human volunteers dosed with a single oral dose of 150 mg OP or an *i.v*. infusion of 150 mg OC. The human model was further validated based on the experimental data of humans receiving multiple oral doses of 50, 100, 200 or 500 mg of OP *b.i.d*. for one week.

#### Statistical and sensitivity analyses

Mean absolute prediction error (MAPE) was used as a measure of good fit between model-predicted concentration (*C*_predi_) and experimental concentration (*C*_expti_). It was calculated using the following equation:


(1)

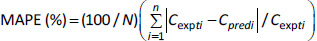



where, i represents individual data points; *N* is the total number of data points. A deviation within a factor of two between predicted and experimental concentration data (*i.e*., MAPE<50 %) was used as the criteria for goodness of fit [38].

Log-normalized sensitivity parameter (LSP) was used to identify key model parameters that had significant impacts on model prediction. LSP was defined as the partial derivatives of model response to the corresponding model parameter [39]:


(2)

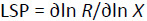



where *R* is the model output and *X* is the parameter for which the sensitivity is assessed. The sensitivity analysis was conducted by AcslXtreme 2.0.1.2 (Aegis Technologies Group, Inc., Huntsville, AL) using the central difference method. The LSP represented the percentage change in an output value associated with the percentage change in the input parameter. The plasma and lung OC concentrations were chosen as the target tissues for model responses following oral administration of 10 mg/kg of OP to rats. Model parameters were changed individually by 0.1 % to assess their impact on model prediction of plasma and lung concentrations. For each model response, magnitude, time dependency, and the mathematical sign of each sensitivity coefficient were examined. The ranking of effects of input parameters on the model prediction was based on the absolute magnitude of the sensitivity coefficient at the time [40]. Generally, the parameters that have large absolute sensitivity coefficients often cause sensitive model outputs in response to a small change in their values because they have a high probability for prediction error to occur due to inaccuracy of the parameter values.

## Results

### Development and validation of rat PBPK model

Table 2 summarizes the final parameter values used to implement the rat and human PBPK models. The oral bioavailability factor (*F*) of OP was about 80 % for both rats and humans. Plasma OP clearances (CL_p_) for humans and rats were 100 L/h and 0.4 L/h, respectively. If the CL_p_ values were normalized by their respective body weights, the CL_p_ of humans (1.4 L/kg/h) and rats (1.6 L/kg/h) were nearly identical. In contrast, plasma OC clearances (CL_c_) of rats and humans were very different even after they were normalized by the body weight; the CL_c_ were 18 L/h for 70kg BW human adult (0.26 L/kg/h) and 0.38 L/h for 0.25 kg BW rat (1.5 L/kg/h), respectively (Table 2). The *K*_m_ and *V*_max_ values of OP biotransformation were 61.2 mg/h (245 mg/kg/h) and 300 mg/L for rats, and 2.6x10^5^ mg/h (3714 mg/kg/h for 70 kg BW) and 76 mg/L for humans.

### OP/OC concentration-time profiles in plasma/tissues of rats

**Figure 3.**
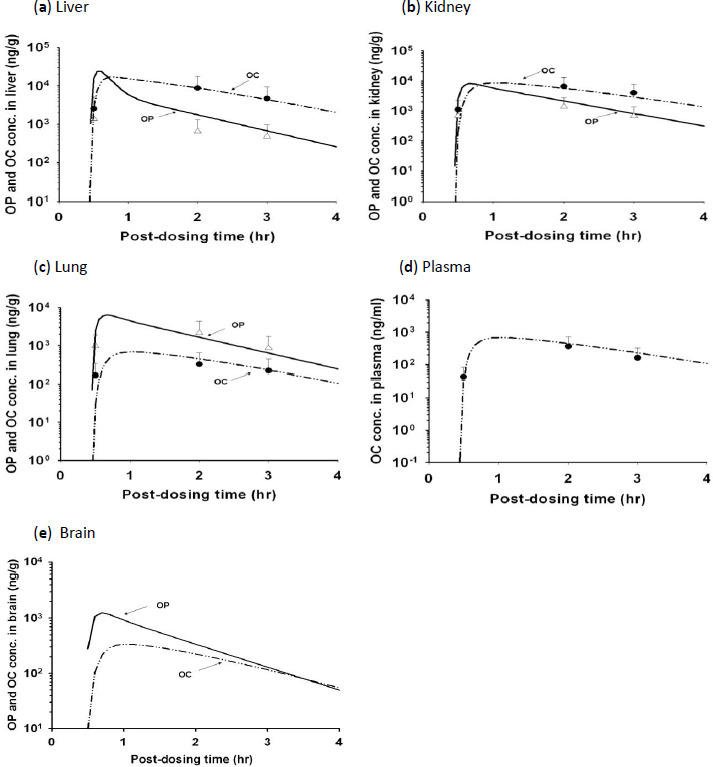
Time course of OP and OC concentrations in the plasma and tissues of rats after administering a single oral dose of 10 mg/kg OP. (**a**) liver, (**b**) kidney, (**c**) lung, (**d**) plasma, (**e**) brain. Data points, means + S.D. of the experimental plasma/tissue concentrations from three different rats; (——) model-simulated OP concentrations; (— - - —) model-simulated OC concentrations

**Figure 4.**
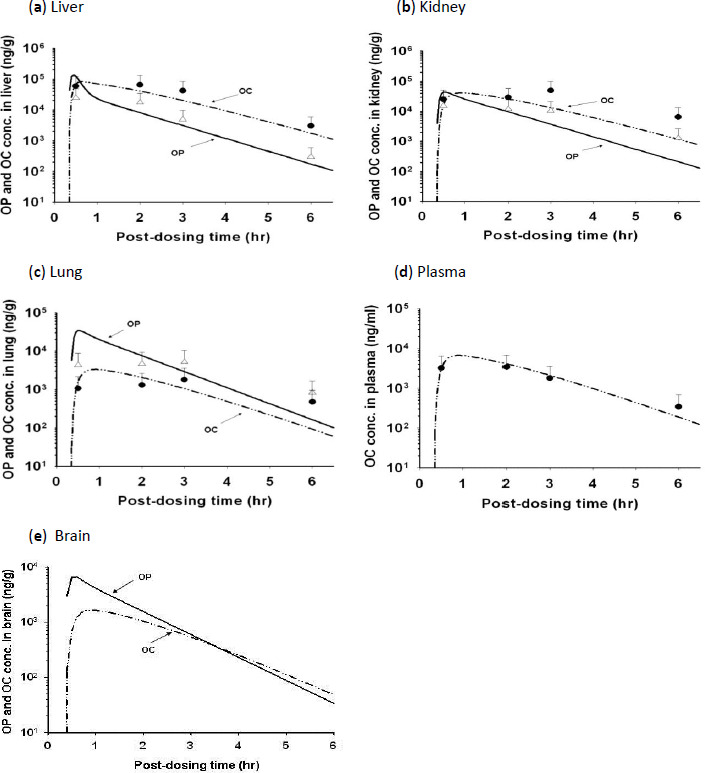
Time course of OP and OC concentrations in the plasma and tissues of rats after administering a single oral dose of 50 mg/kg OP. (**a**) liver, (**b**) kidney, (**c**) lung, (**d**) plasma, (**e**) brain. Data points, means + S.D. of the experimental plasma/tissue concentrations from three different rats; (——) model-simulated OP concentrations; (— - - —) model-simulated OC concentrations

OP was metabolized primarily to OC in rats. Thus, mostly OC and occasionally low concentrations of OP were found in the plasma (Figs. 3 and 4) although both OP and OC were found in the major organs of rats. The *C*_max_ of OC in the plasma were 0.80 and 5.63 μg/ml, respectively after receiving 10 mg/kg and 50 mg/kg of OP by the rat. It decreased in the order of liver > kidney > lung > plasma (Figs. 3 and 4). Although OP was found at a higher concentration than OC in the lung (Figs. 3 and 4) and spleen (data not shown), OP concentration was lower than OC concentration in kidney, liver (Figs. 3 and 4), and muscle (data not shown). The AUC_0-4h_ of OC in the lungs were 5.2 and 26.1 μg·h/ml, respectively when rats were treated with an oral dose of 10 mg/kg and 50 mg/kg OP. These results showed that the dose of OC delivered to the lungs increased in proportion to the OP dose applied externally, a finding which was consistent with the assumption of linear kinetics in the PBPK model.

**Figure 5.**
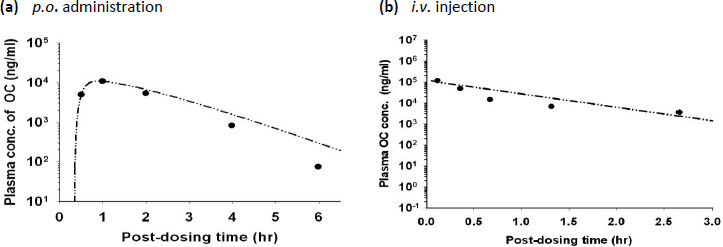
Plasma OC concentration-time profiles in rats after administering (**a**) a single oral dose of 30 mg/g OP, (**b**) an *i.v.* injection of 30 mg/kg OC. Data points, means + S.D. of the experimental plasma/tissue concentrations. (— - - —) model-simulated OC concentrations. Experimental data were adapted from Eisenberg et al. [37]

The rat PBPK model was developed based on the experimental data of the 10 mg/kg treated rats (Fig. 3). It was subsequently validated using the experimental data of the 50 mg/kg treated rats (Fig. 4). In order to fit the experimental data well, however, the *K*_a_ and *F* values of the PBPK model for the 50 mg/kg treated rats had to be higher than those of the 10 mg/kg treated rats, suggesting that OP absorption was more rapid and extensive in the 50 mg/kg treated rats than the 10 mg/kg treated rats (Table 2). The rat PBPK model was also validated using the plasma OC concentration-time data reported by Eisenberg et al. [37]. The rat PBPK model was able to predict accurately the experimental data (Fig. 5) and the tissue distribution data of the 10 mg/kg-treated (Fig. 3) and 50 mg/kg-treated rats (Fig. 4). The PBPK model also was used to simulate the kinetic profiles of OP/OC in the rat’s brain (Figs. 3e and 4e). For rats dosed with 10 mg/kg OP, the predicted *C*_max_ for OP and OC in the brain were 1.25 μg/g and 0.34 μg/g, respectively. For rats dosed with 50 mg/kg OP, the predicted *C*_max_ for OP and OC were 6.64 μg/g and 1.65 μg/g, respectively. These results were in agreement with the other rat tissue/organs in which OP/OC tissue doses were found to increase linearly with the applied OP dose (Figs. 3 and 4). We also performed route-to-route extrapolation by comparing model simulation with the experimental data reported by Eisenberg et al. [37]. The PBPK model was able to describe closely the plasma OP/OC concentration-time profiles of rats receiving either a single dose of 30 mg/kg OP orally or an *i.v.* infusion of 30 mg/kg OC (Fig. 5).

### Sensitivity analysis of rat PBPK model

The sensitivity analysis provided a quantitative means to evaluate how the various input parameters might affect the OC dose metrics in the lungs and plasma when the rats were treated with a single dose of 10 mg/kg OP orally. Table 3 shows a summary of the sensitivity analysis results. The sensitivity coefficients were a mix of positive and negative values which varied with time over the 12 h post-dosing period. We arbitrarily grouped the parameters into 3 different categories according to their absolute sensitivity coefficients: high (>0.5), medium (0.2-0.5), and low (<0.2). Based on these groupings, *F* and *T*_lag_ were classified as high impact parameters, *K*_m_ and *V*_max_ were classified as medium impact parameters, and CL_c_, CL_p_, *K*_a_ and BLPLR_p_ were classified as low impact parameters for the simulated OC dose metrics in the plasma and lungs. Interestingly, BLPLR_c_ had a high impact on the plasma dose metric but a low impact on the pulmonary dose metric. Because the *F* value was positive over the 12 h post-dosing period, OC concentrations in the lung and plasma would increase with an increasing *F* value. Because both of the CL_c_ and CL_p_ sensitivity coefficients had negative values, OC concentrations in the lung and plasma would decrease with increasing CL_c_ and CL_p_ values.

### Calibration of the human PBPK model

**Figure 6.**
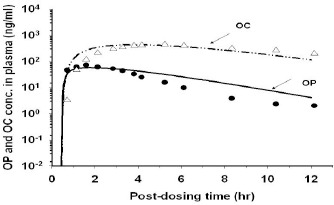
Typical OP and OC concentration-time profiles in the plasma of human volunteers receiving an oral dose of 150 mg OP. Data points represent means + S.D. of the experimental data which are adapted from He et al., [14]. (——) model-simulated OP concentrations; (— - - —) model-simulated OC concentrations

**Figure 7.**
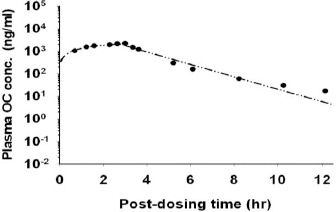
A typical OC concentration-time profile in the plasma of human volunteers receiving an *i.v.* infusion of 150 mg OC. Data points represent means + S.D. of the experimental data which are adapted from He et al., [14]. (— - - —) model-simulated OC concentrations

The human PBPK model was calibrated using the plasma concentration-time data from humans receiving either a single oral dose of 150 mg OP or an *i.v.* infusion of 150 mg OC [14]. Fig. 6 shows the observed concentrations together with the simulated concentrations for humans after *p.o.* administration of OP. Fig. 7 shows the model simulation and the experimental concentration-time profiles of plasma OC in humans during and after a 3-hour *i.v.* infusion of 150 mg OC. The PBPK model was able to describe the experimental data for both administration routes reasonably well. The optimized *F* value for human model was 0.75 (Table 2) which showed extensive absorption of orally administered OP. Simulated *C*_max_ for OP and OC in the plasma was 0.08 and 0.44 μg/ml, respectively, after dosing humans with 150 mg OP orally (Fig.6). The time to reach peak plasma concentration (*T*_max_) for OP and OC was 1 and 4 h, respectively. These results indicated that OC could remain in the plasma for a longer period than OP. Following oral administration of OP, plasma OC concentrations in humans were always >40 ng/ml after the initial absorption phase (Fig. 6), which was much higher than the minimum IC_50_ of neuraminidase inhibition levels (3-10 ng/ml) reported previously by Davies [26].

### Prediction of tissue/plasma OP/OC concentrations in humans receiving multiple OP dose regimens

**Figure 8.**
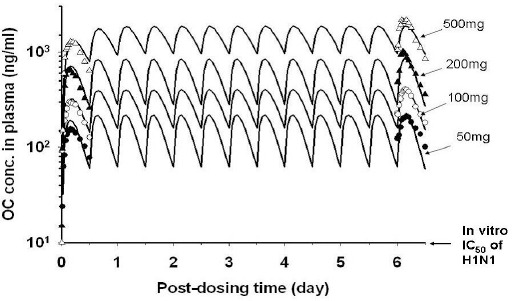
Plasma OC concentration-time profiles in human volunteers receiving multiple oral dose regimens of 50, 100, 200 or 500 mg OP *b.i.d.* for 6 days. Data points represent means + S.D. of the experimental plasma OC concentrations which are adapted from He et al, (17). (——) model-simulated OC concentrations

The PBPK model was used to simulate the kinetics of plasma OC in humans after receiving multiple oral dose regimens of OP [14]. In their studies, the human volunteers were dosed with 50, 100, 200 and 500 mg OP *b.i.d.* for 6 days. The model-predicted *C*_max_/*C*_min_ ratios for OC in the plasma were 3.5 (218 ng/ml : 62 ng/ml), 2.5 (400 ng/ml : 158 ng/ml), 3.0 (838 ng/ml : 279 ng/ml) and 1.9 (1846 ng/ml : 959 ng/ml), respectively (Fig. 8). The *C*_max_/*C*_min_ ratio decreases with an increasing external OP dose except for the 100 mg *b.i.d* dose, which indicates less OC concentration fluctuation in the plasma at a higher OP dose. In addition, the PBPK model was used to predict the internal tissue dose of OC in the brain and the lung of these studies. The steady-state OC doses/concentrations predicted for the lungs and brain were found to be lower than that of the plasma. When the dose metrics of OC were expressed in AUC_0-6day_, they were 21.4, 42.5, 85.3 and 210.2 μg/ml·h, respectively, for the 50, 100, 200, and 500 mg doses. The corresponding OC dose metrics for the lungs were 10.7, 21.2, 42.6, 105.0 μg/ml·h, respectively. For the brain, they were 5.3, 10.6, 21.3, 52.5 μg/ml·h, respectively. Thus, the dose metrics of OC in these organs/tissues generally decrease in the order of plasma > lung > brain although they were all linearly related to the externally applied OP dose.

**Figure 9.**
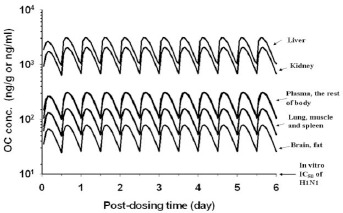
Simulated OC concentration-time profiles in the plasma and tissues of a hypothetical 70-kg human receiving 75 mg OP b.i.d. for 6 days. (——) model-simulated OC concentrations

**Figure 10.**
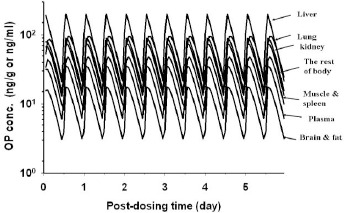
Simulated OP concentration-time profiles in the plasma and tissues of a hypothetical 70-kg human receiving 75 mg OP b.i.d. for 6 days(——) model-simulated OP concentrations

We have also used the human PBPK model to simulate OC (Fig. 9) and OP (Fig. 10) dose metrics in a hypothetical human after receiving the recommended dose regimen of 75 mg OP *b.i.d.* for 6 days. Fig. 9 shows the simulated OC concentrations in the plasma and major organs, which decreases in the order of liver > kidney > plasma ≈ the rest of body > lung > brain ≈ fat. The mean OC concentrations predicted for the lung and brain were ≥0.052 μg/g and ≥ 0.026μg/g, respectively. Fig 10 shows the simulated OP concentrations in the human plasma/organs which decreased in the order of liver > lung > kidney > the rest of body > muscle ≈ spleen > plasma > brain ≈ fat. The predicted mean OP concentrations in the gut was high (3.72 μg/g) indicating a significant amount of OP was not absorbed.

## Discussion

Previous studies have shown rats and humans metabolize OP differently under *in vitro* incubation conditions; OP is hydrolyzed primarily to OC by the carboxylesterases in rat plasma but is not hydrolysed by the carboxylesterases in human plasma [23]. Also, OP is metabolized by the hepatic CYP450 system of rats to minor metabolites [13]. In contrast, although OP is metabolized to OC by the hepatic carboxylesterases in humans, the CYP450 system does not seem to involve in its biotransformation [14].Thus, although OP and OC are found in the plasma of humans after administering OP orally [14,32], only OC and occasionally OP are detected in the plasma of rats (Figs. 3 and 4). The species difference in OP metabolism may be related to the different types of carboxylesterases in rats and humans [41]. As a result, not all systemic OP in humans are converted to OC by the hepatic carboxylesterases [14,32].

Results of our study show the kinetics of *in vitro* OP biotransformation in rat plasma can be adequately described by the Michaelis-Menten equation (Table 2). Because carboxylesterase activity varies among different animal species and organ sites [41], OP biotransformation is assumed to take place only in the liver of rats during PBPK model development (Fig. 2) although the *K*_m_ (300 mg/L) and *V*_max_ (61.2 mg/h) of the rat PBPK model have been derived from rat plasma incubation studies. In contrast, the *K*_m_ (187 μM, 76mg/L) and *V*_max_ (114 nmol/mg/min, 2.6 x 10^5^ mg/h) of the human PBPK model have been obtained from *in vitro* human liver microsomal studies of Shi et al., [2] (Table 2).

Our studies also show that the *C*_max_ of OC in the organs/tissues of rats decreases in the order of liver > kidney > lung > plasma > brain. Similar orders of radioactivity distribution were observed in the ferrets [14] and mice [15] after oral administration of radiolabeled OP. However, because the radioactivity is comprised of a mixture of OP, OC and other metabolites, it is not possible to quantify OP and OC individually based on the results of the tissue distribution studies.

Our PBPK model provides a means to integrate relevant *in vitro* and *in vivo* data into a coherent description of OP/OC pharmacokinetics for the whole animal. The model is comprised of an OP and an OC sub-models (Fig. 2). Model performance is evaluated by comparing simulation results with experimental data. The proposed PBPK model is quite complex but can be collapsed into a less complex model by consolidating the various tissues/organs into rich and poor perfusion compartments. We have chosen a more complex model for the present study because it possesses the structural details to address the pharmacological and toxicological effects of OP in humans. In addition, although it is more difficult to fit available experimental data to a complex model, the derived parameter values from a complex model are more accurate than those derived from a less complex model [19].

More than 80 parameter values are required to implement the PBPK model of OP/OC. There is always uncertainty regarding whether they actually represent true parameter values. We have used the analytical sensitivity analysis [39] to identify the model parameters that significantly affect the OC dose metrics in the lungs and plasma. As shown in Table 3, none of the parameter has an absolute sensitivity coefficient significantly greater than 1.0. This indicates that there is no amplification of errors from the input parameters in the prediction of OC dose metrics in these target organs. In other words, the OC dose metrics in the lungs and plasma are predicted with a certain degree of confidence even though some of the model parameter values may have been determined inaccurately.

The PBPK model in rat was developed majorly with the dataset following orally administered 10 mg/kg, among all the parameters, distribution, metabolism and elimination parameters were basically from experimental data while absorption related parameters were allowed to be optimized to a certain extent, the model could predict the tissue distributions satisfactorily for 10 mg/kg and two published rat plasma datasets [15,16]. While the developed model was used to simulate the tissue exposure in rat following orally administered 50 mg/kg for validation, the simulations seemed to have the tendency of overestimating *C*_max_ and underestimating AUC for both OP and OC in kidney and lung, the discrepancy might be related to the higher optimized parameters related to absorption (*K*_a_, *F*) and renal clearance of OP (CL_p_) compared to the reported values. Considering the satisfactory simulations for exposure at 10 mg/kg and the uncertainty of absorption processes, this leaves space for further refinement or validation. On the other hand, the current PBPK model will be definitely improved with further specific parameters for metabolism considering different enzymes and happening sites between human and rat, absorption/distribution parameters related to P-GP transporters (brain/GI tract) [42-44] and elimination parameters related to organic anion transporters (OAT) in kidney [45].

The therapeutic effects of OP in humans are related to the inhibition of neuraminidase activity of the influenza virus. In other words, inhibition of neuraminidase activity by OC will result in a decrease or even stoppage of progeny virion release and propagation in the respiratory tract. Thus OP efficacy may be evaluated by comparing the dosimetry of OC in the lung and the IC_50_ of OC of neuraminidases. He et al., [14] showed that the IC_50_ of OC for neuraminidases range from 0.3-2.0 nM (0.08-0.28 ng/ml) in an enzymatic assay to 0.6-155 nM (0.17-32.8 ng/ml) in a viral replication inhibition assay. As such the IC_50_ of OC differs among the subtype of influenza virus neuraminidases; it may range from 0.01-3.3 nM and 0.7-2.2 nM for H2N3 and H1N1 influenza viruses, respectively [32]. As shown in Fig. 9, the steady-state concentrations of OC in the plasma/organs of a hypothetical human receiving the recommended OP dose regimen are much higher than the minimum of *in vitro* IC_50_ of H1N1 virus neuraminidase (3-10 ng/ml) [14]. Also, the trough (*C*_min_) of the OC concentration-time curve in the lung is more than 10 fold higher than the IC_50_ value (Fig. 9). As such, OP is an effective anti-viral drug for H1N1 virus and perhaps, for other influenza virus strains as well [32]. The dosimetry of OC in the lung also explains why a dose regimen higher than 75 mg OP *b.i.d.* for one week would not increase the efficacy of OP significantly [3, 4]. As shown above, pulmonary OC concentration already is much higher than the minimum of *in vitro* IC_50_. Thus, a dose regimen higher than 75 mg OP *b.i.d*. is unlikely to have additional inhibitory effects on neuraminidase activity.

The side effects of OP/OC also are explainable by the dose metrics of OP and OC in the brain and gastrointestinal tract; no threshold doses of toxicity have been reported in these organs. As shown in Fig. 8, modeled OC AUC_brain_/AUC_plasma_ ratios for humans dosed with 50, 100, 200, and 500 mg OP are 5.34/21.4, 10.6/42.5, 21.3/85.3, and 52.5/210.2, respectively, (Fig.8) yielding a mean OC AUC_brain_/AUC_plasma_ ratio of about 0.25. Jhee et al [42] have reported that the mean spinal fluid/plasma concentration ratio for OC in humans is 2.93 + 4.06 which is about 10-fold higher than the modeled AUC_brain_/AUC_plasma_ ratio observed in the present study. Other study reported the brain-to-plasma exposure ratios approximately 0.2 for OP and 0.01 for OC [43, 44], these result suggested significant variation between different studies. In our model, the brain/ plasma partition coefficients for OP and OC were 0.5 and 0.25 respectively. It is assumed that only a small amount of OC seems able to penetrate the blood-brain barrier to reach the brain tissue considering OC with high polarity and OP as the substrate of efflux transporter-P-glycoprotein. As such, OP/OC probably is not the major cause of the observed neuropsychiatric effects in humans. As for the gastrointestinal side effects (e.g., abdominal pain, diarrhea, and nausea) [3,4], they may be related to the high levels of OP in the digestive system and contact site toxicity since OP concentrations in the gastrointestinal tract and liver may be as high as 3.72 μg/g and 2.00 μg/g, respectively (Fig. 8).

This is the first study in which a PBPK model is used to predict the internal tissue doses of OP/OC in humans. The human and rat PBPK models are able to predict the pharmacokinetic behaviors and internal tissue doses of OP and OC accurately. Thus, the PBPK model is a valuable, quantitative tool for route-related (Fig. 5), species-related (Fig. 6), and dose-related (Fig. 8) extrapolation. The proposed PBPK model has many practical applications: it can be used to assess the dosimetry of OP/OC in the internal tissues/organs of humans providing a more realistic and accurate account of the dose-response relationship of OP/OC than the externally applied dose. The PBPK model also is useful in adjusting the dose regimens for patients in disease states such as renal failure and liver damage [22].

## Figures and Tables

**Table 1.** Physiological parameters of the OP/OC PBPK model in rats and humans*

Parameter	Organ weight (% of body weight)		Regional blood flow (% of cardiac output)		Partition coefficients	
**Tissue**	**Rat**	**Human**	**Rat**	**Human**	**OP**	**OC**
Adipose	7.6	12	7	5		
Brain	0.57	0.2	2	12		
***Lumped brain***	***8.13***	***12.2***	***9***	***17***	***0.5***	***0.25***
Heart	0.33	0.47				
Lung	0.5	0.76				
***Lumped lung***	***0.83***	***1.23***			***2.5***	***0.5***
Kidney	0.73	0.44	14.1	19	5	8
Liver	3.66	2.57	32.6	25	3	5
GI tract	2.7	1.7	13.1	17	10	1
Muscle	40.4	40	27.8	17	1.4	0.5
Spleen	0.2	0.26	2	2	1.5	0.5
The rest of body^**^	43.4	41.6	16.5	22	3	1
**Total**	100	100	100	100		

^*^Mean data on tissue blood flow rate and volume were adapted from Luttringer et al. [25]. Organ weight was based on the mean BW of a 0.26 kg rat and a 70 kg human; Cardiac output for a 0.25 kg rat is 4.99 L/h; it is calculated from the equation, 14.1*BW^0.75^ (L/h). Cardiac output for a 70 kg human is 390 L/h; it is calculated from the equation, 16.1*BW^0.75^ (L/h).

^**^The brain was lumped into the adipose tissues to form the “brain compartment”; the lung was lumped into the heart to form the “lung compartment”; the remaining organs/tissues including the bone, skin, eye, etc., were grouped together to form “the rest of body”.

**Table 2.** Pharmacokinetic parameters for the OP PBPK model of rats and humans

**Chemical specific parameters**				
Molecular weight	oseltamivir phosphate salt (OP):410.4 oseltamivir carboxylate (OC): 284.35		Used in model	
Unbound fraction in plasma	OP: 53.7 % (rat); 57 (human); OC: no data (rat); 97 % (human)		Not used in model, for reference	
**PK parameters**				
	**Rat**		**Human**	
Parameter	Experimental	Optimized	Experimental	Optimized
*F*	0.35	0.8-0.86	0.75	0.75
*T*_lag_ (h)	NA	0.32-0.44	NA	0.44
*K*a (1/h)	0.76	12-15	NA	0.35 (0.14-0.28)^***^
BLPLR_p_	1	1	NA	1
BLPLR_c_	1	1 (0.7) **	NA	1 (0.6)**
CL_c_ (L/h)	0.38	0.38 (0.5) **	18	18(30)**
CL_p_ (L/h)	NA	0.4	20	100
*K*_m_ (mg/L)	300	300	76.0****	76.0****
*V*_max_ (mg/h)*	61.2	61.2	2.6 x 10^5^ ****	2.6 x 10^5^ ****

*V_max_ was based on a 0.25 kg rat and a 70 kg human

**Bracketed parameter values were used for the *i.v.* route of administration

***Bracketed parameter values were used for multiple oral administration; they were obtained by optimizing the plasma concentration-time data from He et al. [14].

****Shi et al [2], *K*_m_ =187 μm and *V*_max_=114 nmol/mg/min in this paper, the final *V*_max_ was calculated with 70 kg BW, 1.8 kg of liver weight and 52.5 mg microsomes/gram of liver for a standard human adult.

NA: no result.

**Table 3.** Normalized sensitivity coefficients for OC concentrations in the lung and the plasma based on the simulated data of administering 10 mg/kg OP *p.o.* to the rat*

**Pulmonary OC concentration-time data as the model output surrogate**									
		**Time (h)**							
	**Parameter**	**0.5**	**1**	**2**	**4**	**6**	**8**	**10**	**12**
High (>0.5)	*F*	0.03	0.67	0.44	0.11	0.02	-**	-	-
	*T*_lag_	-0.59	-0.03	0.12	0.04	0.01	-	-	-
Medium	*V*_max_	0.03	0.50	0.25	0.03	-	-	-	-
	*K*_m_	-0.03	-0.49	-0.25	-0.03	-	-	-	-
Low (<0.2)	Cl_c_	-	-0.09	-0.18	-0.09	-0.02	-0.01	-	-
	Cl_p_	-	-0.02	-0.06	-0.04	-0.02	-	-	-
	*K*_a_	0.03	0.03	-0.02	-0.01	-	-	-	-
	BLPLR_c_	0***	0	0	0	0	0	0	0
	BLPLR_p_	0	0	0	0	0	0	0	0
**Plasma OC concentration-time data as the model output surrogate**									
High (>0.5)	BLPLR_c_	-0.03	-0.68	-0.45	-0.11	-0.02	-	-	-
	*F*	0.03	0.67	0.44	0.11	0.02	-	-	-
	*T*_lag_	-0.59	-0.03	0.12	0.04	0.01	-	-	-
Medium	*V*_max_	0.03	0.50	0.25	0.03	-	-	-	-
	*K*_m_	-0.03	-0.49	-0.25	-0.03	-	-	-	-
Low (<0.2)	CL_c_	-	-0.09	-0.18	-0.09	-0.02	-0.01	-	-
	CL_p_	-	-0.02	-0.06	-0.04	-0.01	-	-	-
	*K*_a_	0.03	0.03	-0.03	-0.01	-	-	-	-
	BLPLR_p_	0	0	0	0	0	0	0	0

* The sensitivity coefficients were determined using the parameter values from rats dosed with a single oral dose of 10 mg/kg OP (Table 2)

**Absolute value ≤0.001

***: 0 means a change in parameter value did not result in any significant effect on model outputs

## Appendix

The mass balance equations for non-eliminating organs and tissues such as lung, brain, spleen, muscle, and the rest of body are as follows:

(1a)

(1b)

where XY represents the lung, brain, spleen, muscle, and the rest of body. The terms *Q*_XY_, *V*_XY_ and *R*_XY_ represent tissue blood flow, volume and tissue/plasma partition coefficient, respectively; subscripts p and c represent OP and OC, respectively.

### Brain

(2a)

(2b)

### Lung

(3a)

(3b)

### Gut contents

(4a)

(4b)

### Kidney

(5a)

(5b)

### Venous blood

(6a)

(6b)

### Mixed venous plasma

(7a)

(7b)

*C*_PVP_ and *C*_PVC_ are the concentration in mixed venous plasma of OP and OC, *C*_BVP_ and *C*_BVC_ are the concentration in mixed venous blood, BLPLR is the concentration ratio in blood and plasma.

### Arterial blood

(8a)

(8b)

### Liver

(9a)

(9b)

### Gut tissue

(10a)

(10b)

where, *k*_f_ is the fecal excretion rate constant; *V*_max_ and *K*_m_ are the maximum metabolic rate for OP and the constant respectively; *R* = MW_OC_ /MW_OP_, MW is molecular weight; RAO is OP input rate into the blood from the gut, RAO = *k*_a_(*F*)(dose)e^-*k*a(*t*-*t*lag)^, where *k*_a_ represents the absorption rate constant of OP, dose represents OP dosage, *F* represents the apparent or empirical bioavailability factor, and *t*_lag_ represents the lag times for absorption; *k*_f_ is the fecal excretion rate constant; CL_p_ and CL_c_ are the renal clearance for OP and OC, respectively.
